# Interactive effect of obesity and cognitive function decline on the risk of chronic kidney disease progression in patients with type 2 diabetes mellitus: a 9.1-year cohort study

**DOI:** 10.7150/ijms.75824

**Published:** 2022-09-21

**Authors:** Yung-Chuan Lu, Chao-Ping Wang, Wei-Chin Hung, Cheng-Ching Wu, Teng-Hung Yu, Chia-Chang Hsu, Ching-Ting Wei, Fu-Mei Chung, Yau-Jiunn Lee, Wei-Hua Tang

**Affiliations:** 1 Division of Endocrinology and Metabolism, Department of Internal Medicine, E-Da Hospital, Kaohsiung 82445 Taiwan.; 2 School of Medicine for International Students, College of Medicine, I-Shou University, Kaohsiung 82445 Taiwan.; 3 Division of Cardiology, Department of Internal Medicine, E-Da Hospital, Kaohsiung 82445 Taiwan.; 4 School of Medicine, College of Medicine, I-Shou University, Kaohsiung 82445 Taiwan.; 5 Division of Gastroenterology and Hepatology, Department of Internal Medicine, E-Da Hospital, Kaohsiung, 82445 Taiwan.; 6 The School of Chinese Medicine for Post Baccalaureate, College of Medicine, I-Shou University, Kaohsiung, 82445 Taiwan.; 7 Division of General Surgery, Department of Surgery, E-Da Hospital, Kaohsiung, 82445 Taiwan.; 8 Department of Biomedical Engineering, I-Shou University, Kaohsiung, 82445 Taiwan.; 9 Department of Electrical Engineering, I-Shou University, Kaohsiung, 82445 Taiwan.; 10 Lee's Endocrinologic Clinic, Pingtung 90000 Taiwan.; 11 Division of Cardiology, Department of Internal Medicine, Taipei Veterans General Hospital, Yuli Branch, Hualien 98142 Taiwan.; 12 Faculty of Medicine, School of Medicine, National Yang Ming Chiao Tung University, Taipei 112304 Taiwan.

**Keywords:** Chronic kidney disease risk, cognitive function decline, obesity, synergistic interaction, type 2 diabetes mellitus.

## Abstract

**Background:** Obesity and cognitive function decline are independent risk factors for chronic kidney disease (CKD). However, few studies have examined the combined effects of obesity status and cognitive function on change in CKD risk. We aimed to evaluate the association between obesity status, cognitive function and CKD risk change in patients with type 2 diabetes mellitus (T2DM).

**Methods:** Data on 3399 T2DM patients were extracted from a diabetes disease management program between 2006 and 2018. Univariate and multivariate analyses were used to assess the association between obesity, cognitive decline, and CKD risk change. Three indexes, including the relative excess risk of interaction (RERI), attributable proportion of interaction (API), and synergy index (SI), were used to analyze interactions. CKD risk was classified according to the KDIGO 2012 CKD definition.

**Results:** In multivariate analysis, the hazard ratio (HR, 95%Cis) for CKD risk progression was 1.34 (1.12-1.61) times higher in the moderate and severely obese patients compared with the normal weight patients, and 1.34 (1.06-1.67) times higher in the patients with a Mini-Mental State Examination (MMSE) score ≤18 compared to those with an MMSE score ≥24. There was a synergistic interaction between moderate and severe obesity and MMSE score ≤18 on CKD risk progression (SI=4.461; 95% CI: 1.998-9.962), and the proportion of CKD risk progression caused by this interaction was 52.7% (API=0.527; 95% CI: 0.295-0.759). However, normal weight and MMSE score ≥24 were not beneficial on CKD risk improvement in the patients with a moderate risk and very high-risk stage of CKD.

**Conclusion:** There may be a synergistic interaction between obesity and cognitive function decline, and the synergistic interaction may increase the risk of CKD progression.

## Introduction

Type 2 diabetes mellitus (T2DM) is the leading cause of chronic kidney disease (CKD) worldwide. Due to the high risk of progression to end-stage renal disease (ESRD), poor prognosis of morbidity and mortality, and increasing number of patients, CKD has emerged as a global public health burden 1,2. Hence, many studies have investigated its epidemiology, preventive actions, risk factors, and treatment plans 3. The most important traditional risk factors for CKD are diabetes mellitus (DM), hypertension, obesity, hypercholesterolemia, smoking, and alcohol use 4. A more comprehensive understanding of the risk factors that can be modified, such as obesity, and their interactions may help to prevent CKD.

Obesity, including overweight, mild obesity, and moderate and severe obesity has been associated with the development of CKD and end-stage renal disease (ESRD) 5,6,7, and a previous study reported that 24%-33% of all cases of kidney disease were associated with obesity 8. In addition, a link between obesity and the progression of CKD has also been reported, and individuals with a higher body mass index (BMI) have a higher risk of developing proteinuria even without renal disease 9,10. Moreover, the association between obesity and the development and progression of CKD has been reported to be independent of underlying nephropathy 11,12. Furthermore, a higher baseline BMI has been reported to be an independent predictor of ESRD after adjusting for baseline comorbidities, such as DM and hypertension 12. Several studies have also reported associations between cognitive impairment and an increased risk of CKD 13-15 and the severity of kidney disease 15. In addition, a longitudinal study reported a bidirectional relationship between obesity and cognitive function in midlife adults 16. Therefore, there may be pathways between obesity and cognitive function decline that have a common effect on CKD, and these pathways may greatly increase the risk of CKD in people with both conditions. However, previous studies have focused on obesity and cognitive decline as independent predictors of CKD 8,9,14,15, and few have assessed the change in CKD risk in individuals with both obesity and cognitive function.

Therefore, the purpose of this study was to evaluate the association between obesity status, cognitive function, and the risk of CKD progression and regression in patients with type 2 DM (T2DM) based on a diabetes disease management program. We hypothesized that individuals with both obesity and cognitive function would have a higher risk of CKD progression and regression than those with independent factors, and that obesity status and cognitive function would have an interaction effect on CKD risk change.

## Materials and methods

### Study population

From January 2006 to October 2018, patients with T2DM consecutively managed at eight diabetes-specific clinics and the Diabetic Clinic of Kaohsiung E-Da Hospital were enrolled and followed up until December 2021. The diagnosis of T2DM was based on World Health Organization criteria 17. In accordance with the diabetes comprehensive management program covered by the National Health Insurance system in Taiwan, the patients were followed up at 3-month intervals. Each patient underwent standardized physical examinations and biochemical measurements after fasting during the follow-up period, and measurements of urine albumin and urine creatinine were performed within a period of 3 months. All participants received treatment based on standard strategies for hypertension, hyperlipidemia, and diabetes during the follow-up period.

To obtain a comprehensive overview of the change in CKD risk stage and its relationship with obesity status and cognitive function, patients were included if they were age over 18 years, patients with T2DM who had been followed for at least 3 years, and for whom the baseline body mass index (BMI) and Mini-Mental State Examination (MMSE) score had been recorded. A total of 3928 patients with T2DM who were managed in the comprehensive diabetes care program were collected. The exclusion criteria were as follows: (1) patients with documented type 1 diabetes, (2) patients with cancer, (3) patients with liver or urologic diseases, (4) patients who were hospitalized within 3 months prior to enrollment or during the follow-up period, (5) patients who underwent contrast examinations during the follow-up period, (6) patients taking allopurinol or uricosuric agents for gouty arthritis, (7) women who were pregnant, (8) patients who could not provide complete information regarding demographics and medical history, as well as those with missing MMSE data, and (9) patients persistently showing urinary casts and/or hematuria to avoid the potential development/presence of primary glomerular diseases. The study protocol and procedures were approved by the Ethics Committees of Pingtung Christian Hospital and E-Da Hospital, with a Clinical Trial Approval Certificate from Pingtung Christian Hospital on 16th December 2005 and E-Da Hospital Institutional Review Board number EMRP-108-111 and EMRP-109-109. All experiments were carried out in accordance with the approved guidelines.

### Key measures

The WHO definitions of obesity (BMI ≥30 kg/m^2^) and overweight (BMI: 25 to <30 kg/m^2^) are based primarily on criteria derived from studies involving populations of European origin. It has been suggested that a BMI cut-off value of ≥30 kg/m^2^ may be too high for Asian populations, thereby underestimating associated health risks 18,19. Hence, the Ministry of Health and Welfare of Taiwan uses the following definitions based on local statistics 20: underweight (BMI <18.5 kg/m^2^), normal weight (18.5≤ BMI <24 kg/m^2^), overweight (24≤ BMI <27 kg/m^2^), mild obesity (27≤ BMI <30 kg/m^2^), moderate obesity (30≤ BMI <35 kg/m^2^), and severe obesity (BMI ≥35 kg/m^2^). Obesity status at baseline in the present study was classified according to these definitions.

The cognitive function of all participants was assessed according to the MMSE score at baseline. The MMSE is a standardized, brief, and extensively used method to assess cognitive function 21. The MMSE assesses attention, orientation, language, the ability to follow simple verbal and written commands, and immediate and short-term recall. Because it has been shown to be related to age and level of education 22, in the present study, the MMSE score ranges from 0 to 30, with a higher score indicating better cognitive function based on age and level of education. The scores were calculated by the coordinating office staff in this study 23. To investigate the impact of cognitive function on the relationship between obesity status and change in CKD risk, the change in CKD risk was stratified by MMSE score as: MMSE score ≥24, MMSE score 19-23, and MMSE score ≤18 24.

Renal function (estimated glomerular filtration rate (eGFR)) was calculated using the CKD-EPI two-concentration race equation 25: GFR = 141 × min(S_cr_ /κ, 1)^α^ × max(S_cr_ /κ, 1)^-1.209^ × 0.993^Age^ × 1.018 [if female] × 1.159 [if black], where S_cr_ is serum creatinine (mg/dL), κ is 0.7 for females and 0.9 for males, α is -0.329 for females and -0.411 for males, min indicates the minimum of S_cr_/κ or 1, and max indicates the maximum of S_cr_/κ or 1. Albuminuria was measured from spot urine using the albumin-to-creatinine ratio, and the presence of albuminuria was defined by at least two measurements of albumin-to-creatinine ratio >30 mg/g in a 6-month period during follow-up. The CKD risk stage was defined according to eGFR and albuminuria categories following the KDIGO 2012 guidelines 26 as: low risk (eGFR ≥60 mL/min/1.73 m^2^ and urinary albumin-to-creatinine ratio (UACR) <30 mg/g), moderately increased risk (eGFR >60 mL/min/1.73 m^2^ and 30< UACR <300 mg/g, or 45< eGFR <60 mL/min/1.73 m^2^ and 30< UACR <300 mg/g), high risk (30< eGFR <60 mL/min/1.73 m^2^ and UACR >300 mg/g, or eGFR >60 mL/min/1.73 m^2^ and UACR >300 mg/g), and very high risk (15< eGFR <60 mL/min/1.73 m^2^ and UACR >300 mg/g, or eGFR <15 mL/min/1.73 m^2^ and UACR >300 mg/g).

### Laboratory measurements

Routine tests performed during regular follow-up visits included a clinical examination, assessment for any possible adverse reactions to prescribed medicines or diet, body weight, blood pressure, urinary sediment and urinalysis using automated analyzers, complete blood count, serum chemistry, and HbA1c concentration. The urinary albumin concentration was measured after overnight fasting by immunoturbidimetry (Beckman Instruments, Galway, Ireland). The detection limit was 2 mg/L, and the interassay and intraassay coefficients of variance were <8%. In the initial evaluation period, the patients (regardless of duration of diabetes) were defined as being normoalbuminuric if they had a UACR <30 mg/g in at least two consecutive overnight urine collections. During the follow-up period, to confirm the diagnosis of albuminuria, patients with a first UACR measurement >30 mg/g were asked to re-check their urine albuminuria level within 3 to 6 months. Each urine specimen was tested for the presence of urinary infections, and if present, the specimen was discarded and a new sample was collected after treatment. Normal serum creatinine levels (0.8-1.4 mg/dl) and normal urinary sediment (absence of protein, red blood cells, hemoglobin, white blood cells, nitrites and casts) were used to exclude primary renal diseases. Serum creatinine was measured using the Jaffe method. Plasma biochemical parameters and urinary albumin were measured after an overnight fast. Serum HbA1C, total cholesterol, high-density lipoprotein cholesterol (HDL-C), low-density lipoprotein cholesterol (LDL-C), triglycerides, hemoglobin, creatinine, and glucose were determined by standard commercial methods on a parallel-multichannel analyzer (Hitachi 7170A, Tokyo, Japan) as in our previous reports 27,28.

### Variables

The participants underwent face-to-face interviews by trained interviewers using a standard questionnaire that assessed age, sex, cigarette use, and history of diseases (T2DM, diabetes duration, hyperlipidemia, hypertension, heart disease, and cancer). Hypertension was defined as a systolic blood pressure (SBP) ≥140 mmHg, a diastolic blood pressure (DBP) ≥90 mmHg, or if the patient was under antihypertensive treatment. Hyperlipidemia was defined as a triglyceride concentration ≥150 mg/dl, and/or HDL-C <35 mg/dl for men or <39 mg/dl for women, and/or total cholesterol ≥200 mg/dl, and/or LDL-C ≥130 mg/dl, or those undergoing treatment for lipid disorders according to the criteria of the Adult Treatment Panel III. Anthropometric parameters including BMI (kg/m^2^) were measured. Seated blood pressure was measured by a trained nurse with a digital automatic blood pressure monitor (model HEM-907; Omron, Omron, Japan) after the participant had rested for 5 minutes.

### CKD risk change

Participants were followed up for up to 2 years after enrollment. The primary end point was CKD risk change. CKD risk change status (date and causes of CKD risk change) was monitored through the hospital's computerized medical records system and contact with primary physicians. CKD risk change was defined as: stable, the CKD risk stage did not change; progression, the CKD risk stage progressed (i.e. to a moderately increased risk, high risk, or very high risk); and regression, the CKD risk stage improved to a low risk CKD stage. CKD risk change time was calculated as the number of years from the baseline assessment until one of the following: CKD risk change, or end of the study observation period (October 2018, at a maximum of 19 years).

### Statistical analysis

Data normality was analyzed using the Kolmogorov-Smirnov test. Continuous, normally distributed variables are presented as mean ± SD, and non-normally distributed variables as median (interquartile range). Categorical variables are presented as frequencies and/or percentages. Baseline characteristics were compared between groups using one-way ANOVA for normally distributed variables. The chi-square test was used to compare categorical variables. As there were differences in the baseline characteristics, unpaired Student's *t*-tests were used to clarify the differences in each value of physical and metabolic factors between groups.

In this study, we not only investigated the progression to advanced CKD (i.e. moderately increased risk to very high risk) in the patients who had a low risk of CKD, but also regression to a low risk of CKD in the patients who had advanced CKD (moderately increased risk and very high risk) at the time of enrollment. Hazard ratios (HRs) and corresponding 95% confidence intervals (CI) were calculated using univariate and multivariate Cox proportional hazard models to assess the relationships between obesity status and MMSE score with the change in CKD risk. A p value <0.05 was considered to be statistically significant. Furthermore, the proportional hazard assumption was tested graphically using a plot of the log cumulative hazard, where the logarithm of time is plotted against the estimated log cumulative hazard calculated as ln [-ln (S(t))] 29. If the curves for the four obesity groups and the three MMSE score groups were approximately parallel, the proportional hazard assumption was deemed reasonable. All data were analyzed using JMP version 7.0 for Windows (SAS Institute, Cary, NC, USA) and SPSS version 21.0 for Windows (SPSS Inc., Chicago, IL, USA).

In addition, data from an Excel sheet provided by Anderss and co-authors 30 were entered into a database, and relevant indicators of interactions were computed. The value obtained from the Cox regression model was taken as the estimated additive interaction between obesity status and MMSE score. The interaction based on the additive model was evaluated using three indexes, namely the relative excess risk of interaction (RERI), attributable proportion of interaction (API), and synergy index (SI) 31 and their 95% CIs using the delta method 32. The RERI is the excess risk attributed to an interaction relative to the risk without exposure. The API refers to the attributable proportion of disease caused by an interaction in subjects with both exposures. The SI is the excess risk from both exposures when there is a biological interaction relative to the risk from both exposures without an interaction. The RERI has been recommended as the best measure of interaction using a proportional hazards model 33. In the absence of additive interactions, the RERI and AP are equal to 0 34. Indicative biological interactions would be considered when RERI >0, AP >0, or S >1.

## Results

### Cohort description

**Figure 1:**
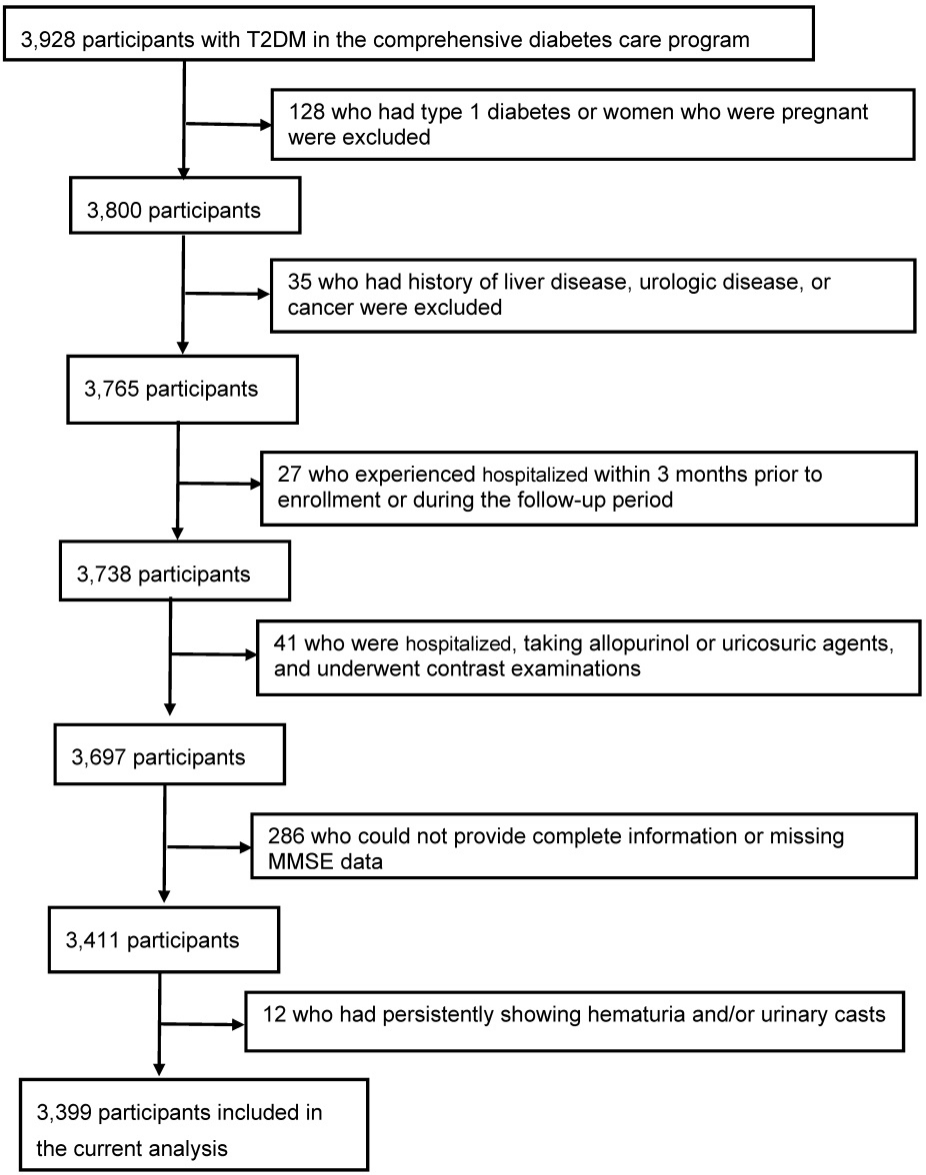
Flow of participants in the comprehensive diabetes care.

Among 3928 consecutive T2DM patients, 529 patients were excluded from the study: 128 who had type 1 diabetes or women who were pregnant, 35 who had history of liver disease, urologic disease, or cancer, 27 who experienced hospitalized within 3 months prior to enrollment or during the follow-up period, 41 who were hospitalized, taking allopurinol or uricosuric agents, and underwent contrast examinations, 286 (7.3%) who could not provide complete information or missing MMSE data, and 12 who had persistently showing hematuria and/or urinary casts. The final study population included 3399 patients (1539 men and 1860 women; age, 72 ± 7 years) (Figure 1).

### General characteristics of the participants

The general characteristics of the 3399 patients grouped according to the change in CKD risk after a mean 9.1 ± 5.0 years are reported in Table 1. There were 1805 (53.1%), 1179 (34.7%), and 415 (12.2%) patients in the stable, progression, and regression groups, respectively. There were no significant differences in sex, normal weight, mild obesity, and moderate obesity rates among the three groups. However, there were significant differences among the three groups in terms of age, diabetes duration, hypertension, hyperlipidemia, smoking status, overweight, severe obesity, MMSE score ≥24, MMSE score 19-23, MMSE score ≤18, all CKD risk stages (low, moderate, high, and very high), eGFR, UACR, creatinine, BMI, SBP, DBP, HbA1c, fasting glucose, total cholesterol, triglycerides, HDL-C, LDL-C, uric acid, medications for T2DM, statins, and angiotensin converting enzyme inhibitors or angiotensin receptor blockers (ACEIs/ARBs). Furthermore, compared with the stable group, the progression group were older, had a longer diabetes duration, and higher rates of hypertension, hyperlipidemia, smokers, moderate obesity, severe obesity, MMSE score 19-23, MMSE score ≤18, moderately increased risk and high risk CKD stages, BMI, SBP, DBP, HbA1c, fasting glucose, total cholesterol, triglycerides, LDL-C, uric acid, and prescriptions of medications for T2DM, statins, and ACEIs/ARBs, and lower rates of overweight, MMSE score ≥24, low risk and very high risk CKD stages, and HDL-C. Moreover, compared with the progression group, the regression group had higher rates of MMSE score ≥24, moderately increased risk, high risk, and very high risk CKD stages, UACR, and creatinine, a younger age, and lower rates of hypertension, MMSE score 19-23, MMSE score ≤18, low risk CKD stage, eGFR, SBP, uric acid, and prescriptions of medications for T2DM.

### Association between obesity status and MMSE score in relation with CKD risk progression

We used univariate and multivariate Cox proportional hazard models to investigate associations between both obesity status and MMSE score in relation to a moderately increased and very high risk CKD stage at follow-up (Table 2). In univariate Cox regression analysis, the baseline clinical and biochemical variables associated with the risk of CKD progression were age, DBP, HDL-C, LDL-C, triglycerides, and fasting sugar in all patients. In multivariate Cox regression analysis, DBP, HDL-C, LDL-C, triglycerides, and fasting sugar were confirmed to be independent factors for the risk of CKD progression after adjustments for age and sex (multivariate model 1). Individuals who were overweight and mildly obese did not have an increased risk of CKD progression compared to those who had a normal weight in all three of the models. Patients who were moderately and severely obese had an increased risk of progressing to a moderately high and very high risk stage of CKD compared to those with a normal weight in all three of the models (HR: 1.25, 95% CI: 1.05-1.49, p=0.011, HR: 1.29, 95% CI: 1.09-1.54, p=0.004, and HR: 1.34, 95% CI: 1.12-1.61, p=0.001, respectively). In addition, individuals with an MMSE score 19-23 did not have an increased risk of progressing to a moderately high and very high risk stage of CKD compared to those with an MMSE score ≥24 in any of the models. Individuals with an MMSE score ≤18 had an increased risk of progressing to a moderately high and very high risk stage of CKD compared to those with an MMSE score ≥24 in all three of the models (HR: 1.31, 95% CI: 1.05-1.62, p=0.018, HR: 1.30, 95% CI: 1.03-1.62, p=0.026, and HR: 1.34, 95% CI: 1.06-1.67, p=0.012, respectively) (Table 2).

### Association between obesity status and MMSE score in relation with CKD risk progression stratified by sex

When the patients were stratified by sex, in male patients who were moderately and severely obese had an increased risk of progressing to a moderately high and very high risk stage of CKD compared to those with a normal weight in model 3 (HR: 1.33, 95% CI: 1.04- 1.69, p=0.026) (Supplementary Table 1). In female patients who were moderately and severely obese had an increased risk of progressing to a moderately high and very high risk stage of CKD compared to those with a normal weight in all three of the models (HR: 1.30, 95% CI: 1.04-1.62, p=0.022, HR: 1.32, 95% CI: 1.06-1.64, p=0.015, and HR: 1.37, 95% CI: 1.09-1.72, p=0.008, respectively). Furthermore, in the female patients, individuals with an MMSE score ≤18 had an increased risk of progressing to a moderately high and very high risk stage of CKD compared to those with an MMSE score ≥24 in model 1 and model 3 (HR: 1.32, 95% CI: 1.02-1.69, p=0.038, HR: 1.32, 95% CI: 1.01-1.71, p=0.043). However, in the male patients, individuals with an MMSE score ≤18 did not have an increased risk of progressing to a moderately high and very high risk stage of CKD compared to those with an MMSE score ≥24 in all three of the models (Supplementary Table 1).

### Association between obesity status and MMSE score in relation with CKD risk improvement

We also used univariate and multivariate Cox proportional hazard models to investigate associations between both obesity status and MMSE score in relation with CKD risk improvement at follow-up (Table 3). In univariate Cox regression analysis, the baseline clinical and biochemical variables associated with CKD risk improvement were age, SBP, DBP, HDL-C, and LDL-C in all patients. In multivariate Cox regression analysis, smoking, SBP, DBP, HDL-C, and LDL-C were confirmed to be independent factors for CKD risk improvement after adjustments for age and sex (multivariate model 1). However, there were no significant associations between obesity status (normal weight, overweight, and mild obesity) and MMSE score (MMSE score ≥24 and MMSE score 19-23) and improvement in the risk of CKD with moderate and severe obesity or MMSE score ≤18 as references in all three of the models, except for normal weight in multivariate model 1 (adjusted for age, sex) and MMSE score ≥24 in univariate Cox regression (Table 3).

### Association between obesity status and MMSE score in relation with CKD risk improvement stratified by sex

When the patients were stratified by sex, in the male patients, there were no significant associations between obesity status (normal weight, overweight, and mild obesity) and MMSE score (MMSE score ≥24 and MMSE score 19-23) and improvement in the risk of CKD with moderate and severe obesity or MMSE score ≤18 as references in models 1 to 3 (Supplementary Table 2). In the female patients, we found that normal weight was significantly related to CKD improvement in model 2 and model 3 (HR: 1.63, 95% CI: 1.12-2.40, p=0.010, HR: 1.51, 95% CI: 1.02-2.25, p=0.039). However, there were no significant associations between MMSE score (MMSE score ≥24 and MMSE score 19-23) and improvement in the risk of CKD with MMSE score ≤18 as references in models 1 to 3 (Supplementary Table 2).

### Interaction of obesity with cognitive decline on the risk of CKD progression

The additive interaction terms of obesity status and MMSE score were constructed, including normal weight and better cognitive function (MMSE score ≥24), better cognitive function and moderate and severe obesity, cognitive decline (MMSE score ≤18) and normal weight, moderate and severe obesity and cognitive decline (MMSE score ≤18) (Table 4). The results showed that the risk of CKD progression in patients with both moderate and severe obesity and cognitive decline was 2.01 times (HR=2.01; 95% CI: 1.25-3.05, p=0.005) higher than that in those without. In multivariate analysis, the patients with both moderate and severe obesity and cognitive decline had a significantly higher risk of CKD progression than those without after adjusting for age and sex (HR=2.04; 95% CI: 1.26-3.13, p=0.005), and this risk was still present after adjusting for all of the confounders (HR=2.04; 95% CI: 1.24-3.18, p=0.006; Table 4). Cox regression analysis showed that the interactive indexes in the three models [single factor (univariate model), adjusted for age and sex (multivariate model 1), and adjusted for all confounders (multivariate model 2)] were as follows: RERI (univariate model: 1.540; 95% CI, 0.110-3.190), RERI (multivariate model 1: 1.571; 95% CI, 0.150-3.293), RERI (multivariate model 2: 1.645; 95% CI, 0.242-3.530); API (univariate model: 0.518; 95% CI, 0.298-0.739), API (multivariate model 1: 0.525; 95% CI, 0.301-0.749), API (multivariate model 2: 0.527; 95% CI, 0.295-0.759); SI (univariate model: 4.565; 95% CI, 1.929-10.804), SI (multivariate model 1: 4.723; 95% CI, 1.993-11.196), SI (multivariate model 2: 4.461; 95% CI, 1.998-9.962). The 95% CIs of the RERI and API suggested that there may be a synergistic interaction between moderate and severe obesity and cognitive decline on CKD risk progression.

### Sensitivity analysis

**Figure 2:**
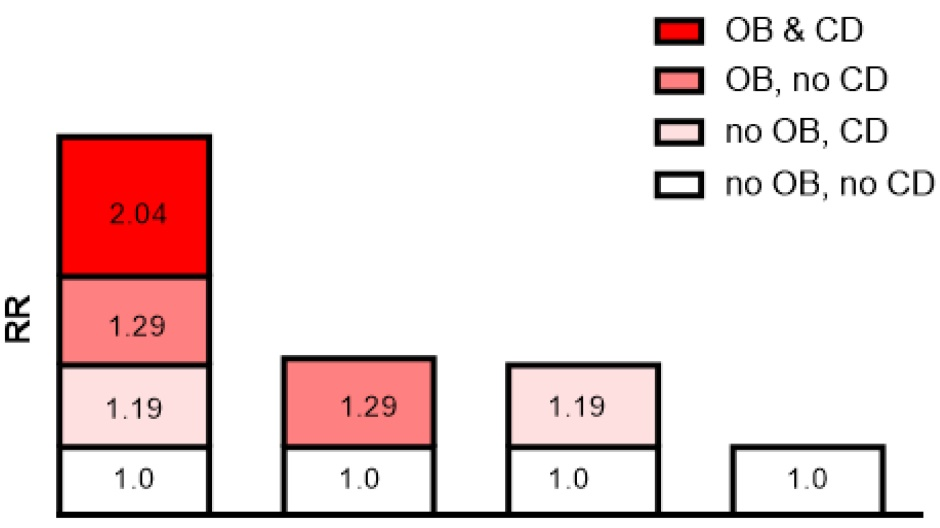
Interaction schematic diagram between moderate and severe obesity (OB) and cognitive decline (CD) on risk of chronic kidney disease risk progression after adjusting for multiple confounders.

In addition, the API was 0.527 after adjusting for all confounders, indicating that the proportion of CKD risk progression that may have been caused by the interaction of moderate and severe obesity and cognitive decline was 52.7% in all CKD risk progression patients. Detailed results are shown in Table 4 and Figure 2.

## Discussion

In this study, we evaluated the associations between obesity, cognitive function decline and change in CKD risk in patients enrolled from a diabetes disease management program. Our results showed that obesity and cognitive decline were independent risk factors for CKD progression, and that obesity and cognitive decline may have a synergistic interaction in the progression of CKD risk. In addition, the interaction of obesity and cognitive decline accounted for 52.7% of the risk of progression in the patients with a low risk CKD stage. With regards to obesity status, only moderate and severe obesity may have interacted with cognitive decline in the progression of CKD risk.

An association between obesity or cognitive decline with CKD has been reported in many studies 5,6,13-15. In this study, the risks of CKD progression in the individuals with moderate and severe obesity and cognitive decline were 1.34 times and 1.34 times those of the individuals with a normal weight and MMSE score ≥24, respectively. Our results are generally consistent with previous studies which assessed the association of obesity 12 or cognitive decline 15 with the risk of CKD progression. In addition, Hartanto et al. found a bidirectional association between obesity and cognitive function in midlife adults 16. Both obesity and cognitive decline have been identified as risk factors for CKD, however their joint effects on CKD risk progression have rarely been studied. Our results revealed that the patients with both moderate and severe obesity and cognitive decline had a 2.04 times higher risk of CKD progression than those with a normal weight and MMSE score ≥24 after adjusting for all confounders, showing a possible synergistic interaction between obesity and cognitive decline on CKD risk progression. In addition, our results suggest that only moderate and severe obesity may interact with cognitive decline on CKD risk progression. However, further prospective clinical studies are needed to verify these findings.

The interaction of moderate and severe obesity with cognitive decline on CKD risk progression may be explained by biological and behavioral pathways. There are several common mechanisms in the associations between obesity, cognitive decline and CKD risk progression, including inflammation, metabolic disorders, and endothelial dysfunction 35-40. With regards to inflammation 35,36, obesity can be mediated by downstream comorbid conditions such as hypertension or DM. However, adiposity can also affect the kidneys directly, and endocrine activity of adipose tissue can produce leptin, visfatin, resistin, and other adipokines 41-43 to promote the formation of an inflammatory microenvironment 35,44, which has also been related to cognitive decline and CKD risk progression 36,38. In addition, obesity and cognitive decline in patients with CKD risk progression are both related to metabolic disorders. Yun et al. reported that the increased risk of CKD progression from obesity may be associated with metabolic abnormalities 45. Metabolic disorders have also been associated with a higher risk of developing cognitive impairment 46. This association can be moderated by additional factors such as oxidative stress, genetic factors, pro-inflammatory processes, lifestyle, age, and education, which are also thought to contribute to CKD risk progression. In addition, obesity is associated with macro- and microvascular endothelial dysfunction. Microvascular endothelial dysfunction has also been reported to be a significant risk factor for cognitive impairment 40, and it may be a mechanism for an increased risk of CKD progression 47. Three possible mechanisms have been proposed to explain the impact of the interaction between obesity and cognitive decline on CKD risk progression 35-40. However, further studies are needed to verify these mechanisms.

In the present study, normal weight and better cognitive function (MMSE score ≥24) were not associated with an improvement in CKD risk in the patients with a moderately high risk and very high risk stage of CKD. However, due to the limited number of cases, we cannot draw firm conclusions about the possible effect of normal weight and better cognitive function on CKD risk regression. Few studies have explored the relationship between normal weight or weight loss and the future risk of renal disease. Ramirez et al. found a J-shaped association between the prevalence of proteinuria and BMI 47, and Reynolds et al. reported a J-shaped association between BMI and the risk of ESRD 48. In addition, Ryu et al. reported an increased risk of CKD in patients who lost <-0.75 kg/year in both overweight and normal weight groups 49. In contrast, another study reported that weight loss among obese individuals without overt renal diseases was associated with an improvement in glomerular hemodynamic abnormalities 50. In addition, a cross-sectional study by Småbrekke et al. did not find evidence of an association between low-grade cognitive impairment on either the kidneys or brain in a middle-aged general population 51. Therefore, further studies are needed to clarify the role of normal weight or better cognitive function on the change in CKD risk.

In the present study, when the patients were stratified by sex, in the female patients, individuals with an MMSE score ≤18 had an increased risk of progressing to a moderately high and very high risk stage of CKD compared to those with an MMSE score ≥24 (Supplementary Table 1). However, in the male patients, individuals with an MMSE score ≤18 did not have an increased risk of progressing to a moderately high and very high risk stage of CKD compared to those with an MMSE score ≥24 (Supplementary Table 1). It is not surprise that the results of MMSE score ≤18 for the increased risk of CKD progressing were differences between men and women. In previous studies, female has higher risk of CKD progression, especially in diabetic elderly women; it might due to hormonal change, sex-specific genetic polymorphism, and the higher prevalence of dyslipidemia, hypertension, and obesity in female compared to the male counterparts 52,53. Similarly, in our study, we found that female patients had higher BMI than those of male patients (26.2 ±4.1 kg/m^2^ vs. 25.9±3.5 kg/m^2^, p = 0.016). More interesting is in our study, when the patients were stratified by sex, in the male patients, there were no significant associations between obesity status (normal weight, overweight, and mild obesity) and improvement in the risk of CKD with moderate and severe obesity as references. In the female patients, we found that normal weight was significantly related to CKD improvement (Supplementary Table 2).

This study has some limitations. First, MMSE score was the only measure of cognitive function, and we cannot rule out that using an extensive battery of neuropsychologic assessments may have yielded different results. However, MMSE score has been widely used in validation studies in a hospital setting 54,55. Second, BMI may not be an ideal marker of obesity, because high BMI does not differentiate patients with relatively high bone mass or muscle mass who are not truly obese, and other indices, such as waist-to-height ratio or waist circumference, have been suggested to be better markers of obesity 56. In addition, previous study showed that higher BMI was not associated with deficits in episodic memory and executive functions 57. Nevertheless, in clinical practice, BMI are easier to be measured, so it remains the predominant index to establish obesity; hence, our results have direct clinical relevance. Third, our findings were derived from Chinese subjects, and thus they may not be generalizable to other ethnic populations. Fourth, the underlying biochemical and biophysiological mechanisms underlying our observations should be investigated. Whether other clinical serum markers such as known inflammatory markers and uremic toxins 58,59 are involved in CKD risk progression in patients with moderate and severe obesity combined with cognitive decline should also be clarified.

## Conclusions

This study demonstrated that obesity and cognitive function decline were independent risk factors for CKD risk progression, and that there may be a synergistic interaction between moderate and severe obesity and cognitive decline on CKD risk progression in patients with T2DM. In addition, there may be common pathways between obesity and cognitive decline leading to the CKD risk progression. However, further prospective clinical studies are needed to further validate our results and elucidate the mechanisms underlying these results. In addition, normal weight and MMSE score ≥24 were not beneficial with regards to CKD risk improvement in the T2DM patients with moderately increased risk and very high risk stages of CKD. Further investigations are warranted to investigate the potential mediators contributing to these findings.

## Supplementary Material

Supplementary figure and tables.

## Figures and Tables

**Table 1:** Baseline clinical and biochemical characteristics of the study participants stratified by chronic kidney disease risk change followed for a mean 9.1 ± 5.0 years (N=3399).

	Stable	Progression	Regression	p value
Number	1805	1179	415	
Age (years)	62.8±7.5	64.4±7.7^a^	63.1±8.4^b^	<0.0001
Sex, female (n, %)	975(54.0)	646(54.8)	239(57.6)	0.418
Diabetes duration (years)	4(1-10)	6(2-11)^a^	4(1-11)	<0.0001
Hypertension (n, %)	1009(55.9)	893(75.7)^a^	285(68.7)^b^	<0.0001
Hyperlipidemia (n, %)	1428(79.1)	997(84.6)^a^	356(85.8)	<0.0001
Smokers (n, %)	373(20.7)	294(24.9)^a^	86(20.7)	0.017
Obesity status (n, %)				
Normal weight	560(31.0)	350(29.7)	133(32.1)	0.602
Overweight	649(36.0)	369(31.3)^a^	130(31.3)	0.017
Mild obesity	360(19.9)	252(21.4)	94(22.7)	0.387
Moderate obesity	212(11.8)	168(14.3)^a^	48(11.6)	0.105
Severe obesity	24(1.3)	40(3.4)^a^	10(2.4)	0.001
MMSE score status (n, %)				
≥24	1596(88.4)	953(80.8)^a^	364(87.7)^b^	<0.0001
19-23	125(6.9)	136(11.5)^a^	32(7.7)^b^	<0.0001
≤18	84(4.7)	90(7.6)^a^	19(4.6)^b^	0.002
CKD risk (n, %)				
Low risk	1203(66.7)	636(53.9)^a^	0(0.0)^b^	<0.0001
Moderate risk	325(18.0)	386(32.7)^a^	253(61.0)^b^	<0.0001
High risk	110(6.1)	157(13.3)^a^	123(29.6)^b^	<0.0001
Very high risk	167(9.3)	0(0.0)^a^	39(9.4)^b^	<0.0001
eGFR (ml/min/1.73m^2^)	79.1±22.8	77.7±20.0	69.8±21.9^b^	<0.0001
UACR (mg/g)	12.1(5.6-31.8)	19.3(9.4-54.8)	49.5(30.8-108.6)^b^	0.003
Creatinine (μmol/l)	79.6(70.7-97.2)	79.6(70.7-97.2)	88.4(70.7-114.9)^b^	<0.0001
Body mass index (kg/m^2^)	25.8±3.7	26.3±4.1^a^	26.0±3.9	0.003
SBP (mmHg)	133±18	138±19^a^	135±19^b^	<0.0001
DBP (mmHg)	77±12	79±12^a^	78±12	<0.0001
HbA1c (%)	8.0±1.8	8.4±2.0^a^	8.3±1.9	<0.0001
Fasting glucose (mg/dl)	150.0±55.6	156.7±61.9^a^	152.3±59.0	0.009
Total cholesterol (mg/dl)	188.5±38.5	191.9±37.9^a^	191.1±40.6	0.049
Triglycerides (mg/dl)	116.0(84.0-163.0)	122.0(87.0-179.0)^a^	133.0(94.0-187.0)	<0.0001
HDL cholesterol (mg/dl)	51.3±13.7	49.2±12.8^a^	50.0±13.6	0.0001
LDL cholesterol (mg/dl)	105.2±33.4	109.1±32.7^a^	106.0±33.1	0.007
Uric acid (mg/dl)	5.4±1.6	6.0±1.7^a^	5.5±1.7^b^	<0.0001
Type of treatment (%)				
(OHA/Insulin/Both)	70.4/2.8/26.8	56.0/5.4/38.6^a^	67.5/1.5/31.1^b^	<0.0001
Statins (n, %)	1401(77.6)	983(83.4)^a^	350(84.3)	<0.0001
ACEI/ARB (n, %)	767(42.5)	770(65.3)^a^	257(61.9)	<0.0001

Patients were included in the stable group if they maintained the same CKD risk stage, in the progression group if the CKD risk stage had progressed, and in the regression group if their condition had improved to a low risk CKD stage. Data are presented as mean ± SD, frequency (percent), or median (interquartile range). MMSE, mini-mental state examination; eGFR, estimated glomerular filtration rate, UACR, urinary albumin-to- creatinine ratio, CKD, chronic kidney disease; SBP, systolic blood pressure; DBP, diastolic blood pressure; HDL, high-density lipoprotein; LDL, low-density lipoprotein; OHA, oral hypoglycemic agents; ACEI/ARB, angiotensin converting enzyme inhibitor or angiotensin receptor blocker. ^a^Significant as compared with the stable group. ^b^Significant as compared with the progression group.

**Table 2:** Hazard ratios (HRs) for the association between obesity, MMSE score, and progression to moderately increased risk and very high risk stage of chronic kidney disease in type 2 diabetic patients with a low risk stage of chronic kidney disease.

	Univariate		Multivariate model 1		Multivariate model 2	
	HR (95% CI)	p-value	HR (95% CI)	p-value	HR (95% CI)	p-value
Age	1.01 (1.00-1.02)	0.020	-	-	-	-
Sex	1.09 (0.97-1.22)	0.154	-	-	-	-
Smoking (yes versus no)	1.13 (0.99-1.29)	0.075	1.13 (0.96-1.32)	0.154	-	-
Systolic blood pressure (per unit)	1.00 (0.99-1.01)	0.062	1.00 (0.99-1.01)	0.099	-	-
Diastolic blood pressure (per unit)	1.01 (1.00-1.01)	0.026	1.01(1.00-1.01)	0.017	-	-
Total cholesterol (per unit)	0.99 (0.99-1.00)	0.517	0.99 (0.99-1.00)	0.664	-	-
HDL-cholesterol (per unit)	0.99 (0.99-1.00)	<0.0001	0.99 (0.99-1.00)	0.002	-	-
LDL-cholesterol (per unit)	1.01 (1.01-1.02)	<0.0001	1.01 (1.01-1.02)	<0.0001	-	-
Triglycerides (per unit)	1.00 (1.00-1.00)	0.012	1.00 (1.00-1.00)	0.010	-	-
Fasting sugar (per unit)	1.00 (1.00-1.00)	<0.0001	1.01 (1.01-1.02)	<0.0001	-	-
Obesity status						
Normal weight	Ref		Ref		Ref	
Overweight	0.94(0.81-1.09)	0.405	0.94(0.81-1.09)	0.383	1.00(0.86-1.17)	0.983
Mild obesity	1.04(0.89-1.22)	0.627	1.06(0.90-1.25)	0.465	1.17(0.99-1.38)	0.071
Moderate and severe obesity	1.25(1.05-1.49)	0.011	1.29(1.09-1.54)	0.004	1.34(1.12-1.61)	0.001
MMSE score						
≥24	Ref		Ref		Ref	
19-23	1.15(0.96-1.38)	0.126	1.15(0.95-1.38)	0.164	1.17(0.97-1.42)	0.095
≤18	1.31(1.05-1.62)	0.018	1.30(1.03-1.62)	0.026	1.34(1.06-1.67)	0.012

Multivariate model 1: Adjusted for age, gender; Multivariate model 2: Adjusted for age, gender, smoking status, systolic blood pressure, diastolic blood pressure, total cholesterol, high-density lipoprotein cholesterol, low-density lipoprotein cholesterol, triglycerides, and fasting sugar. HDL-C, high-density lipoprotein cholesterol; LDL, low-density lipoprotein; HR, hazard ratio; CI, confidence interval.

**Table 3:** Hazard ratios (HRs) for the association between obesity status, MMSE score, and chronic kidney disease risk improvement to low risk stage in type 2 diabetic patients with moderately increased risk and very high risk stage of chronic kidney disease.

	Univariate		Multivariate model 1		Multivariate model 2	
	HR (95% CI)	p-value	HR (95% CI)	p-value	HR (95% CI)	p-value
Age	0.95 (0.94-0.97)	<0.0001	-	-	-	-
Sex	0.98 (0.81-1.19)	0.828	-	-	-	-
Smoking (yes versus no)	0.83 (0.65-1.05)	0.125	0.70 (0.53-0.93)	0.014	-	-
Systolic blood pressure (per unit)	0.99 (0.98-0.99)	<0.0001	0.99 (0.98-1.00)	0.0001	-	-
Diastolic blood pressure (per unit)	0.99 (0.98-0.99)	0.049	0.99 (0.98-1.00)	0.007	-	-
Total cholesterol (per unit)	0.99 (0.99-1.00)	0.275	0.99 (0.99-1.00)	0.118	-	-
HDL-cholesterol (per unit)	1.01 (1.01-1.02)	0.0003	1.01 (1.01-1.02)	0.0003	-	-
LDL-cholesterol (per unit)	0.99 (0.99-1.00)	0.0002	0.99 (0.99-1.00)	<0.0001	-	-
Triglycerides (per unit)	1.00 (0.99-1.00)	0.111	1.00 (0.99-1.00)	0.286	-	-
Fasting sugar (per unit)	0.99 (0.99-1.00)	0.726	0.99 (0.99-1.00)	0.365	-	-
Obesity status						
Normal weight	1.31(0.97-1.80)	0.078	1.48(1.09-2.03)	0.012	1.28(0.93-1.79)	0.761
Overweight	1.13(0.84-1.56)	0.424	1.26(0.92-1.73)	0.147	1.18(0.86-1.64)	0.847
Mild obesity	1.23(0.89-1.71)	0.214	1.28(0.92-1.78)	0.143	1.18(0.85-1.66)	0.823
Moderate and severe obesity	Ref		Ref		Ref	
MMSE score						
≥24	1.54(1.00-2.52)	0.048	1.16(0.74-1.92)	0.542	1.20(0.75-2.05)	0.652
19-23	0.94(0.54-1.69)	0.838	0.88(0.50-1.58)	0.654	0.91(0.51-1.67)	0.503
≤18	Ref		Ref		Ref	

Multivariate model 1: Adjusted for age, gender; Multivariate model 2: Adjusted for age, gender, smoking status, systolic blood pressure, diastolic blood pressure, total cholesterol, high-density lipoprotein cholesterol, low-density lipoprotein cholesterol, triglycerides, and fasting sugar. HDL-C, high-density lipoprotein cholesterol; LDL, low-density lipoprotein; HR, hazard ratio; CI, confidence interval.

**Table 4:** Cox proportional hazards regression analysis of the interactive items between moderate and severe obesity and cognitive decline on risk of chronic kidney disease risk progression.

	Univariate		Multivariate model 1		Multivariate model 2	
	HR (95% CI)	p-value	HR (95% CI)	p-value	HR (95% CI)	p-value
Age	1.01 (1.00-1.02)	0.020	-	-	-	-
Sex	1.09 (0.97-1.22)	0.154	-	-	-	-
Smoking (yes versus no)	1.13 (0.99-1.29)	0.075	1.13 (0.96-1.32)	0.154	-	-
Systolic blood pressure (per unit)	1.00 (0.99-1.01)	0.062	1.00 (0.99-1.01)	0.099	-	-
Diastolic blood pressure (per unit)	1.01 (1.00-1.01)	0.026	1.01(1.00-1.01)	0.017	-	-
Total cholesterol (per unit)	0.99 (0.99-1.00)	0.517	0.99 (0.99-1.00)	0.664	-	-
HDL-cholesterol (per unit)	0.99 (0.99-1.00)	<0.0001	0.99 (0.99-1.00)	0.002	-	-
LDL-cholesterol (per unit)	1.01 (1.01-1.02)	<0.0001	1.01 (1.01-1.02)	<0.0001	-	-
Triglycerides (per unit)	1.00 (1.00-1.00)	0.012	1.00 (1.00-1.00)	0.010	-	-
Fasting sugar (per unit)	1.00 (1.00-1.00)	<0.0001	1.01 (1.01-1.02)	<0.0001	-	-
**Interactive items between moderate and severe obesity and cognitive decline**						
Normal weight and MMSE ≥24	Ref		Ref		Ref	
Moderate and severe obesity	1.21 (1.00-1.47)	0.046	1.24 (1.02-1.51)	0.033	1.29 (1.04-1.59)	0.019
MMSE score ≤18	1.22 (0.79-1.80)	0.350	1.18 (0.76-1.77)	0.450	1.19 (0.75-1.80)	0.455
Moderate and severe obesity and MMSE ≤18	2.01 (1.25-3.05)	0.005	2.04 (1.26-3.13)	0.005	2.04 (1.24-3.18)	0.006
RERI (95% CI)	1.540 (0.110-3.190) 0.518 (0.298-0.739) 4.565 (1.929-10.804)		1.571 (0.150-3.293) 0.525 (0.301-0.749) 4.723 (1.993-11.196)		1.645 (0.242-3.530) 0.527 (0.295-0.759) 4.461 (1.998-9.962)	
API (95% CI)						
SI (95% CI)						

Multivariate model 1: Adjusted for age, gender; Multivariate model 2: Adjusted for age, gender, smoking status, systolic blood pressure, diastolic blood pressure, total cholesterol, high-density lipoprotein cholesterol, low-density lipoprotein cholesterol, triglycerides, and fasting sugar. HDL-C, high-density lipoprotein cholesterol; LDL, low-density lipoprotein; HR, hazard ratio; CI, confidence interval; RERI, relative excess risk of interaction; API, attributable proportion due to interaction; SI, synergy index.
