# Supplementary material for: Interactive effect of obesity and cognitive function decline on the risk of chronic kidney disease progression in patients with type 2 diabetes mellitus: a 9.1-year cohort study
Source: Int J Med Sci. 2022 Sep 21;19(11):1660–71. doi: 10.7150/ijms.75824 (PMC9553862; doi:10.7150/ijms.75824)
Supplement: Supplementary file 1 — Supplementary figure and tables. [file ijmsv19p1660s1.pdf]

SUPPLEMENTARY DATA

Interactive effect of obesity and cognitive function decline on the risk of chronic kidney disease progression in patients with type 2 diabetes mellitus: a 9.1-year cohort study

Contents

Supplementary Figure 1. Proportionality hazard assumptions for obesity status and MMSE score……………………………………………………………………………………………..2

Supplementary Table 1. Hazard ratios (HRs) for the association between obesity, MMSE score, and progression to moderately increased risk and very high risk stage of chronic kidney disease in male and female type 2 diabetic patients with a low risk stage of chronic kidney disease……………………………………………………………………………………………..3

Supplementary Table 2. Hazard ratios (HRs) for the association between obesity status, MMSE score, and chronic kidney disease risk improvement to low risk stage in male and female type 2 diabetic patients with moderately increased risk and very high risk stage of chronic kidney disease…………………………………………………………………………4

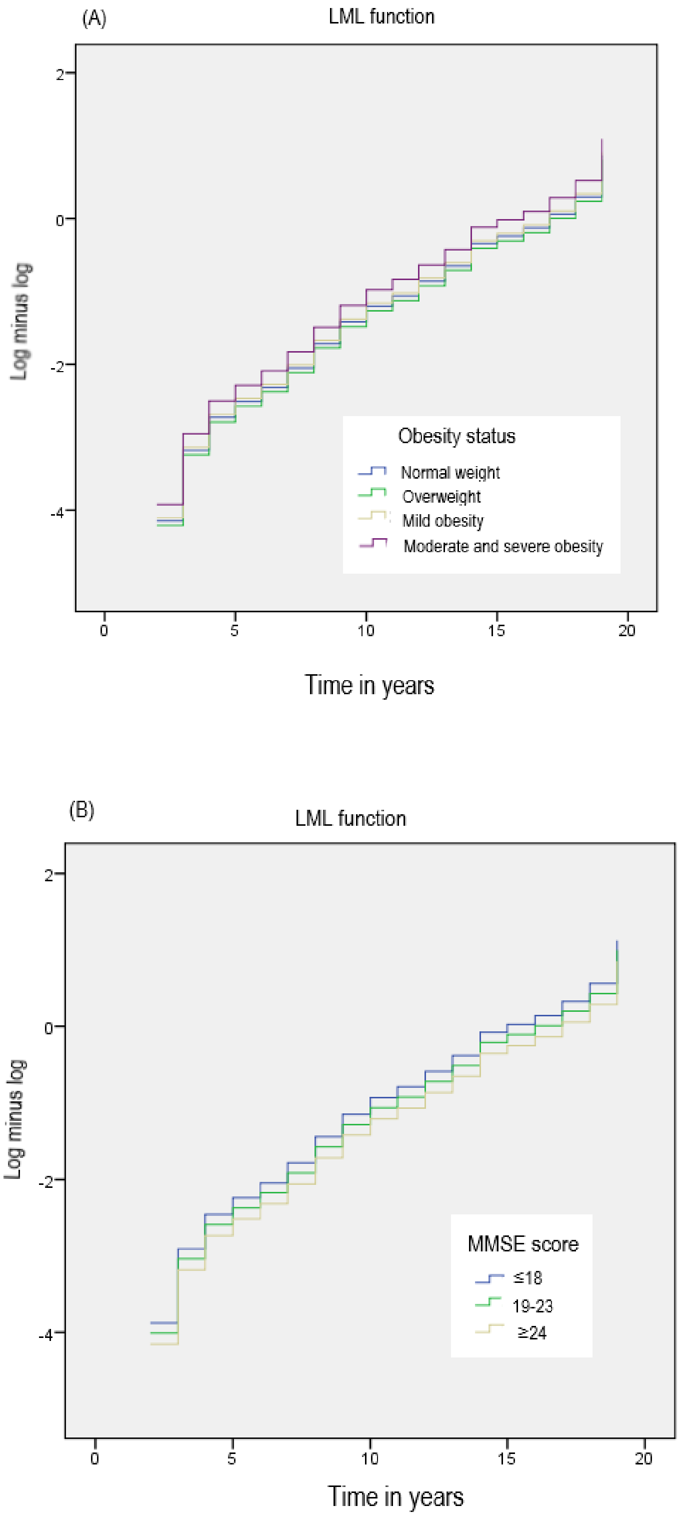

Figure 1. Proportionality hazard assumptions for obesity status (A) and MMSE score (B)

Supplementary Table 1. Hazard ratios (HRs) for the association between obesity, MMSE score, and progression to moderately increased risk and very high risk stage of chronic kidney disease in male and female type 2 diabetic patients with a low risk stage of chronic kidney disease

		Model 1	Model 2	Model 3
Variables		HR (95%CI)	HR (95%CI)	HR (95%CI)
**Male patients**				
Obesity status	Normal weight	1.00 (reference)	1.00 (reference)	1.00 (reference)
	Overweight	0.91(0.73-1.12)	0.91(0.74-1.12)	0.97(0.78-1.21)
	Mild obesity	1.19(0.94-1.51)	1.21(0.95-1.53)	1.32(0.98-1.76)
	Moderate and severe obesity	1.20(0.90-1.59)	1.22(0.92-1.62)	**1.33(1.04-1.69)**[a]
MMSE score	≥24	1.00 (reference)	1.00 (reference)	1.00 (reference)
	19-23	1.35(0.94-1.89)	1.35(0.93-1.89)	1.38(0.95-1.94)
	≤18	1.42(0.90-2.12)	1.41(0.88-2.13)	1.43(0.90-2.17)
**Female patients**				
Obesity status	Normal weight	1.00 (reference)	1.00 (reference)	1.00 (reference)
	Overweight	0.97(0.79-1.19)	0.96(0.78-1.18)	1.01(0.82-1.24)
	Mild obesity	0.94(0.75-1.18)	0.95(0.76-1.18)	1.03(0.82-1.29)
	Moderate and severe obesity	**1.30(1.04-1.62)**[a]	**1.32(1.06-1.64)**[a]	**1.37(1.09-1.72)**[b]
MMSE score	≥24	1.00 (reference)	1.00 (reference)	1.00 (reference)
	19-23	1.13(0.91-1.40)	1.05(0.83-1.31)	1.09(0.87-1.36)
	≤18	**1.32(1.02-1.69)**[a]	1.24(0.95-1.60)	**1.32(1.01-1.71)**[a]

Model 1: Univariate Cox proportional hazards regression analysis; Model 2: Adjusted for age; Model 3: Adjusted for age, gender, smoking status, systolic blood pressure, diastolic blood pressure, total cholesterol, high-density lipoprotein cholesterol, low-density lipoprotein cholesterol, triglycerides, fasting sugar. HR, hazard ratio; CI, confidence interval. [a]$p<0.05$ and [b]$p<0.01$.

Supplementary Table 2. Hazard ratios (HRs) for the association between obesity status, MMSE score, and chronic kidney disease risk improvement to low risk stage in type 2 diabetic patients with moderately increased risk and very high risk stage of chronic kidney disease

		Model 1	Model 2	Model 3
Variables		HR (95%CI)	HR (95%CI)	HR (95%CI)
**Male patients**				
	Normal weight	1.26(0.74-2.27)	1.35(0.80-2.44)	1.22(0.70-2.22)
	Overweight	1.14(0.67-2.04)	1.23(0.73-2.21)	1.17(0.68-2.11)
Obesity status	Mild obesity	1.38(0.79-2.53)	1.43(0.82-2.61)	1.39(0.80-2.56)
	Moderate and severe obesity	1.00 (reference)	1.00 (reference)	1.00 (reference)
MMSE score	≥24	2.03(0.77-8.23)	1.52(0.56-6.25)	1.33(0.49-5.46)
	19-23	1.47(0.43-6.73)	1.29(0.37-5.92)	1.13(0.32-5.19)
	≤18	1.00 (reference)	1.00 (reference)	1.00 (reference)
**Female patients**				
	Normal weight	1.38(0.96-2.03)	**1.63(1.12-2.40)**[a]	**1.51(1.02-2.25)**[a]
	Overweight	1.15(0.79-1.70)	1.29(0.88-1.91)	1.19(0.81-1.76)
Obesity status	Mild obesity	1.14(0.76-1.72)	1.18(0.79-1.79)	1.13(0.75-1.72)
	Moderate and severe obesity	1.00 (reference)	1.00 (reference)	1.00 (reference)
MMSE score	≥24	1.49(0.92-2.57)	1.05(0.64-1.83)	1.05(0.64-1.84)
	19-23	0.84(0.45-1.61)	0.80(0.43-1.54)	0.71(0.37-1.37)
	≤18	1.00 (reference)	1.00 (reference)	1.00 (reference)

Model 1: Univariate Cox proportional hazards regression analysis; Model 2: Adjusted for age, gender; Model 3: Adjusted for age, gender, smoking status, systolic blood pressure, diastolic blood pressure, total cholesterol, high-density lipoprotein cholesterol, low-density lipoprotein cholesterol, triglycerides, fasting sugar. HR, hazard ratio; CI, confidence interval. [a]$p<0.05$.
